# Longitudinal effects of sex differences and apolipoprotein E genotype on white matter engagement among elderly

**DOI:** 10.1093/braincomms/fcaf278

**Published:** 2025-07-17

**Authors:** Hui Zhang, Jingrao Zhang, Chun Liang Hsu, Edward S Hui, Kai-Hei Tse, Henry Ka-Fung Mak, David H K Shum

**Affiliations:** Department of Rehabilitation Sciences, The Hong Kong Polytechnic University, 999077 Hong Kong, China; Department of Rehabilitation Sciences, The Hong Kong Polytechnic University, 999077 Hong Kong, China; Department of Rehabilitation Sciences, The Hong Kong Polytechnic University, 999077 Hong Kong, China; Department of Imaging and Interventional Radiology, The Chinese University of Hong Kong, 999077 Hong Kong, China; Department of Psychiatry, The Chinese University of Hong Kong, 999077 Hong Kong, China; Department of Health Technology and Informatics, The Hong Kong Polytechnic University, 999077 Hong Kong, China; Brain and Mind Centre, University of Sydney, Camperdown, 2050 NSW, Australia; Department of Neuropathology, Royal Prince Alfred Hospital, Camperdown, 2050 NSW, Australia; Department of Diagnostic Radiology, The University of Hong Kong, 999077 Hong Kong, China; Department of Rehabilitation Sciences, The Hong Kong Polytechnic University, 999077 Hong Kong, China

**Keywords:** white matter engagement maps, APOE4, default mode network, memory, sex difference

## Abstract

The apolipoprotein E (*APOE*) ɛ4 allele is the primary genetic risk factor that influences lipid metabolism and contributes to distinctive Alzheimer's disease pathologies, including increased hippocampal atrophy and accelerated cognitive decline. Synaptic dysfunction can occur in *APOE4* carriers even before the appearance of any clinical symptoms. Recent evidence has suggested that this genetic risk factor impacts males and females differently. The sex-specific vulnerability for females to cognitive decline, particularly memory, intensifies post-menopause and emphasizes the need for further investigation. White matter abnormalities, *APOE4* allele and disruptions in default mode network connectivity serve as early indicators that are crucial for better understanding Alzheimer's disease progression. This study aims to explore relationships between biological sex, *APOE4*, default mode network-white matter activity and memory function as measured by the Selective Reminding Test. Participants were categorized by risk level on their *APOE4* status. Using longitudinal data from the Harvard Aging Brain Study, we examined sex differences in default mode network-white matter engagement among older individuals with and without the *APOE4* allele. Our findings demonstrated a significant reduction in default mode network-white matter activity in the right posterior corona radiata in the high-risk group compared to the low-risk group. High-risk females showed reduction in default mode network-white matter activity in the right superior longitudinal fasciculus, which positively correlated with free recall performance, compared to their low-risk counterparts. Unlike females, males showed no significant changes between the low- and high-risk groups. These results underscore the effectiveness of white matter engagement mapping in differentiating longitudinal changes in memory function related to the genetic risk factor *APOE4* and biological sex.

## Teaser

Zhang *et al*. explored white matter involvement in the default mode network, revealing its relevance to memory processes. Correlations between neuroimaging and behavioural data substantiated this approach, enhancing understanding of the apolipoprotein E genotype and emphasizing the importance of sex-specific research in Alzheimer's neural mechanism and progression.

## Graphical Abstract

**Graphical Abstract:**
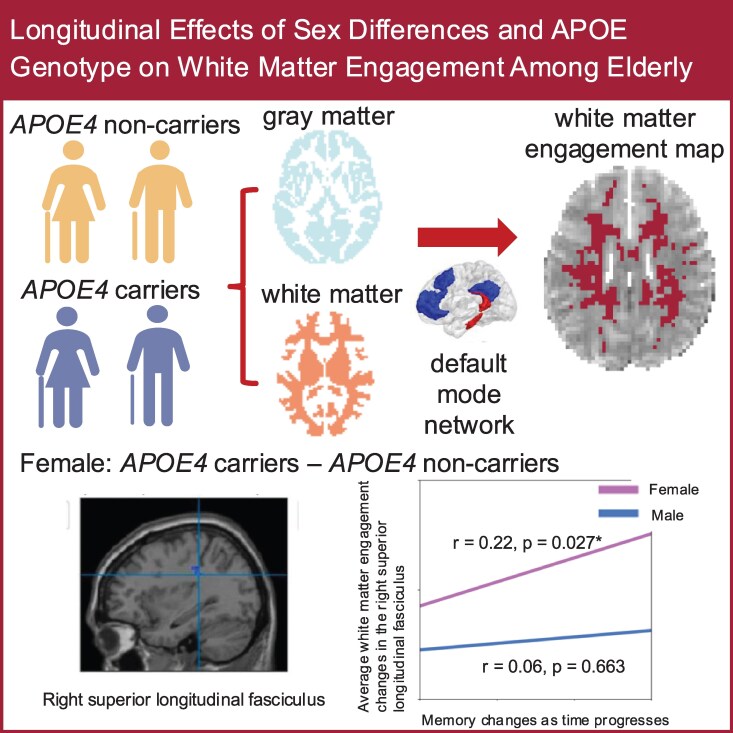

## Introduction

Global populations are aging at an accelerated rate, with the number of individuals over 65 projected to double by 2050.^1^ Alzheimer's disease impacts 3–4% of adults during their late working years or upon entering retirement.^2^ Cognitive decline, particularly in memory, is prevalent in the aging populations at risk of Alzheimer's disease. However, the progression of memory loss varies significantly and is influenced by a combination of genetic and biological factors.

Several studies have identified age, sex and the apolipoprotein E allele 4 (*APOE4*) gene as significant risk factors for memory loss.^3,4^ As the strongest genetic risk factor for Alzheimer's disease,^5^  *APOE4* contributes to accelerated brain changes decades before clinical symptoms emerge, including grey matter (GM) atrophy and synaptic dysfunction.^4,6^ Notably, these effects are sex-dependent. For example, one study examining the *APOE*-by-sex interaction in healthy controls (*n* = 5496) and mild cognitive impairment (MCI) individuals (*n* = 2588) showed that the link between the *APOE4* allele and cognitive decline is stronger in females.^7^ Several studies revealed that females exhibited significantly steeper reductions in hippocampal volume,^8^ white matter (WM) microstructural integrity [fractional anisotropy (FA)]^9^ and more pronounced memory declines compared to males.^8,10^ Findings of these studies underline the critical need to consider the interplay between sex and the *APOE* genotype when assessing their impacts on seemingly healthy brains.

Decrease in WM volume is evident around the age of 40 years as a part of the normal aging process.^11^ In addition to the established clinical biomarkers such as tau or amyloid beta, WM abnormalities are one of the earliest signs of Alzheimer's disease development.^12,13^ For example, myelin content in the frontal gyrus begins to decline in the fourth decade of life.^14,15^ WM changes were particularly pronounced in healthy and asymptomatic individuals carrying the *APOE4* allele compared to non-carriers,^16^ and demyelination accelerates decline in cognitive function, including memory,^17,18^ executive dysfunctions^19^ and negatively affect the default mode network (DMN).^20-22^. Using resting-state functional magnetic resonance imaging (rs-fMRI), previous studies have identified that DMN connectivity significantly varies between cognitively healthy *APOE4* carriers and non-carriers.^23,24^ Importantly, areas of the DMN are central to memory function, including the precuneus,^25^ ventromedial prefrontal cortex,^25^ hippocampus^26^ and lateral temporal lobe.^27^ Among older adults, both DMN connectivity and hippocampal volume are independently associated with memory performance.^27^ Yuan *et al*.^25^ reported that among individuals with *APOE4* and amnestic MCI (aMCI), the anterior and posterior subnetworks of DMN were associated with impaired episodic memory. This suggests that *APOE* polymorphism may influence antagonistic neural pathways within these DMN subnetworks.

The application of diffusion tensor imaging (DTI)^28^ and T_2_-weighted-fluid-attenuated inversion recovery^29^ provides valuable insights into the integrity of WM. Among APOE4-related studies, Honea *et al*.^21^ noted decreases in FA in the parahippocampus and hippocampal atrophy in healthy elderly individuals carrying the *APOE4* allele. Operto *et al*.^22^ found significant differences in the superior longitudinal fasciculus (SLF), which connects the frontal regions to the posterior brain regions (temporal and parietal lobes), between *APOE* carriers and non-carriers. Studies have demonstrated that integrating fMRI and DTI enables exploration of the relationship between WM and the fluctuations in coordinated neural activity essential for cognition.^30,31^ Furthermore, understanding the relationship between functional and structural networks, often referred to as structural connectivity-functional connectivity (SC-FC) coupling, may significantly improve the evaluation of how neurodegenerative disorders manifest.^32,33^ However, direct integration of these imaging techniques is complex and extends beyond a simple linear correspondence because of the differences in biophysical origins.^34^ Instead, a WM engagement map offers a new way to detect and analyse WM signals within the fMRI data set.^35^ Traditionally, WM signals are considered nuisances and are removed during fMRI data pre-processing.^36^ Recent studies suggest that WM blood oxygen level-dependent (BOLD) signals may reflect neural activities.^37,38^ Integrating WM and GM BOLD signals could provide a more precise depiction of the brain's functional architecture.^35^ By applying WM BOLD, insights integrating GM and WM will be invaluable for clinicians in identifying disease progression and implementing effective therapeutic strategies for patients.

In this secondary analysis of longitudinal imaging and blood biomarker data collected from 173 healthy elderly participants from the Harvard Aging Brain Study (HABS),^39^ we aim to uncover the complex interaction between biological sex, *APOE4*, WM engagement with the DMN and memory. We hypothesize that both the *APOE4* allele and biological sex would impact WM engagement with the DMN, where female *APOE4* carriers will be affected the most, as demonstrated by poor WM engagement of the DMN and poor memory performance over time.

## Methodology

### Participants

Demographic information including age, sex, years of education, *APOE* genotyping and neuropsychological testing (Table 1), along with MRI data comprising T_1_, T_2_, fluid-attenuated inversion recovery (FLAIR), susceptibility weighted imaging and rs-fMRI, was obtained from the HABS data set of healthy elderly participants who underwent scans in both the first and fourth years. Informed consent for all participants was obtained by HABS, and our use of the data was approved by HABS.

**Table 1:** Comparison of demographic, descriptive and the non-primary neuropsychological characteristics between high-risk and low-risk groups at two visits

	Visit 1				Visit 2			
Variables	Non-APOE4 (*N* = 121)	APOE4 (*N* = 52)	*t* value	*P* value	Non-APOE4 (*N* = 121)	APOE4 (*N* = 52)	*t* value	*P* value
Age (years)	73.26 ± 6.45	72.91 ± 5.62	0.34	0.733^a^	76.35 ± 6.44	75.99 ± 5.61	0.35	0.728^a^
Sex (F/M)	78/43	31/21	0.37	0.545^b^	78/43	31/21	0.37	0.545^b^
Education (years)	16.08 ± 3.1	16.46 ± 2.71	−0.76	0.446^a^	16.08 ± 3.1	16.46 ± 2.71	−0.76	0.446^a^
Clinical Dementia Rating Scale (score)	0.01 ± 0.06	0.00 ± 0.00	0.93	0.35	0.05 ± 0.15	0.08 ± 0.18	−0.95	0.34
MMSE (score)	29.16 ± 0.95	29.08 ± 0.99	0.50	0.62	29.31 ± 0.94	29.23 ± 0.96	0.53	0.60
Geriatric Depression Scale (score)	2.98 ± 2.67	3.31 ± 2.96	−0.71	0.48	3.51 ± 3.82	4.37 ± 4.92	−1.09	0.28
CAT (*n*)	45.67 ± 10.15	47.5 ± 9.52	−1.11	0.270^a^	44.08 ± 9.61	44.73 ± 9.49	−0.41	0.685^a^
DigitSym (score)	48.48 ± 9.78	49.04 ± 10.02	−0.34	0.735^a^	47.85 ± 10.84	47.13 ± 10.15	0.41	0.686^a^
FAS_total (*n*)	46.5 ± 12.28	46.4 ± 14.07	0.04	0.966^a^	46.77 ± 11.54	46.87 ± 13.13	−0.05	0.961^a^
TMT_(B − A)/A (score)	1.52 ± 1.12	1.37 ± 0.95	0.84	0.402^a^	1.31 ± 0.92	1.44 ± 0.89	−0.86	0.389^a^
VFDT (score)	30.4 ± 2.28	30.71 ± 1.74	−0.87	0.387^a^	30.74 ± 1.98	31.1 ± 1.51	−1.15	0.251^a^
DemoMemQs (score)	1.01 ± 1.11	1.5 ± 1.37	−2.48	0.014^a,^*	1.27 ± 1.53	1.37 ± 1.56	−0.39	0.699^a^

CAT, Category Fluency Test; DigitSym, Digit Symbol Test; FAS_total, total score of Phonemic Fluency; TMT_(B − A)/A, Trail Making Test—difference in time to complete Parts B and A, normalized by time for Part A; VFDT, Visual Form Discrimination Test; DemoMemQ, Structured Telephone Interview of Dementia Assessment.

^a^Two independent samples *t*-test.

^b^ *χ* ^2^ test.

^*^ *P* < 0.05; **P < 0.01.

The HABS included individuals who (i) were 62 years or older; (ii) had a score of 0 on the Clinical Dementia Rating Scale, thereby excluding participants with dementia; (iii) scored above 25 on the Mini-Mental State Examination, ruling out participants with MCI or dementia; (iv) scored above the age- and education-adjusted cut-offs on the 30-min delayed recall on the Logical Memory Story A, identifying those without MCI; and (v) had a score of less than 11 on the Geriatric Depression Scale, indicating the absence of significant depressive symptoms. Exclusion criteria included a history of alcoholism, drug abuse, head trauma or any ongoing serious medical or psychiatric conditions.^39^

For our planned investigation, we only included HABS participants who had *APOE* genotyping at the study baseline and those who had a complete data set at all time points. In the end, altogether, this study included a subset of 173 participants who were followed up in both the first and fourth years.

### Participant grouping

*APOE* status was collected in this cohort.^39^ Participants were categorized based on the presence or absence of the *APOE4* allele. Those without the *APOE4* allele (i.e. those with *APOE* 2/2, *APOE* 2/3, *APOE* 3/3) were assigned to the low-risk group, while those with at least one *APOE4* allele (*APOE* 2/4, *APOE* 3/4, *APOE* 4/4) were placed in the high-risk group.^24^ Subgroup analyses via stratification by biological sex were conducted to separate participants into high-risk female, low-risk female, high-risk male and low-risk male groups.

### Cognitive function variables

Participants in the HABS underwent a comprehensive series of neuropsychological tests. Cognitive domains tested in the HABS included: executive functions [Category Fluency Test (CAT), Phonemic Fluency (FAS) and Trail Making Test (TMT)]; semantic memory [Boston Naming Test (BNT)]; working memory [Digit Span Test and Letter-Number Sequencing Test (LetterNum)]; verbal and visual memory [Selective Reminding Test (SRT), Logical Memory (LogicMem) and Free and Cued Selective Reminding Test (FCsrt)]; speed of information processing and visual coordination (Digit Span Test); visual perception [Visual Form Discrimination Test (VFDT)]; and dementia screening [Structured Telephone Interview for Dementia Assessment (DemoMemQ)]. DemoMemQ consists of seven items that assess subjective memory complaints, yielding a dementia score ranging from 0 to 81, with higher scores indicating greater impairment.^40^

Within the context of the present study, our primary focus was on examining the change in memory function across approximately 3 years. Therefore, the following measurements of memory were selected as the primary outcomes: BNT, Digit Span Test, LetterNum, SRT, LogicMem and FCsrt. The cognitive variables for our primary outcomes are depicted in Table 2.

**Table 2:** Primary cognitive function variables of this study

Cognitive function variables	Description	
Boston Naming Test (BNT)	A measure of semantic memory that evaluates an individual's ability to name objects. It consists of 60 large ink drawings divided into two 30-question versions (even/odd). If an individual is unable to recognize an item, semantic and phonemic cues are provided to assist with identification	
Digit Span Test_Forword minus Backward (Digits_F_B)	The test measures working memory by requiring participants to repeat a sequence of numbers in the same order (forward) or in reverse (backward) as read by an examiner. The test concludes when a participant fails to accurately reproduce a sequence or exceeds the sequence limit (nine digits forward, eight digits backward). In this study, the forward minus backward score is used to assess working memory capacity	
Free and Cued Selective Reminding Test (FCsrt)	This test identifies early-stage dementia by assessing memory retrieval through three trials each of free and cued recall. The final score combines the total images recalled in both the free and cued trials, evaluating the participant's ability to retrieve information spontaneously and with cues	
	FCsrt_FNC	Total of free and cued recall
	FCsrt_Free	Total of free recall
Letter-Number Sequencing Test (LetterNum)	This working memory test involves the examiner reading combinations of letters and numbers, which the participant must reorder and recite—numbers first in ascending order, followed by letters alphabetically. Scoring awards 1 point for each accurately ordered sequence	
Logical Memory (LogicMem)	The LogicMem involves an oral presentation of a short story to the participant, who is then asked to immediately repeat the story as accurately as possible. After a delay of 20–30 min, the participant is asked to recall and recount as many details as they can remember from the story	
	LogicMem_IL	Measure of immediate learning
	LogicMem_DR	Measure of delayed recall
Selective Reminding Test (SRT)	The test evaluates verbal memory through a multiple trial list-learning task. The examiner reads a list of 12 unrelated words, and the participant is asked to immediately recall as many words as possible. On each subsequent trial, the participant is presented with only the words they failed to recall on the previous trial until a total of six trials are completed. After a brief delay, they are asked to produce as many words as they can remember. For those words they cannot retrieve, they must select the correct word from a four-item multiple choice	
	SRT_dr	Delayed recall, assess the ability to recall information after a specified delay period
	SRT_cltr	Continuous long-term retrieval, the number of words recalled over three consecutive trials
	SRT_cr	Continuous retrieval, the number of words consistently retrieved across two consecutive trials
	SRT_ltr	Long-term retrieval, the number of words retrieved from long-term storage
	SRT_lts	Long-term storage, the number of words recalled on two consecutive trials without a reminder between trials
	SRT_mc	Recognition recall, the ability to recognize previously presented words from multiple-choice options
	SRT_str	Short-term retrieval, the number of words retrieved from short-term storage
	SRT_tr	Total recall, the total number of words recalled across all trials

### MRI scanning protocol

Participants in the HABS underwent MRI examinations using a 3 T MR scanner (Siemens Tim Trio) equipped with a 12-channel head coil. Structural images were acquired using a 3D T_1_-weighted sequence, employing magnetization-prepared rapid gradient echo imaging. The parameters of this sequence included 256 sagittal slices, a repetition time (TR) of 2300 ms, an echo time (TE) of 2.95 ms, an inversion time (TI) of 900 ms, a flip angle (FA) of 9 degrees, a field of view (FOV) of 270 × 253 mm^2^, a matrix size of 256 × 240 mm^2^ and a voxel size of 1.05 × 1.05 × 1.2 mm^3^. Additionally, rs-fMRI data were acquired using gradient-echo echo-planar sequence with a TR/TE of 3000/30 ms, a flip angle of 90 degrees, a voxel size of 3 × 3 × 3 mm^3^ and a total of 124 volumes.

### MRI data analysis

The pre-processing of fMRI data was conducted utilizing the Statistical Parametric Mapping (SPM12) software (https://www.fil.ion.ucl.ac.uk/spm/software/spm12/). The initial four volumes were discarded to allow for signal stabilization, and the subsequent fMRI images were then adjusted to account for variations in image acquisition timing. Images from each participant were realigned using a six-parameter (rigid body) linear transformation. Participants exhibiting head movements exceeding 3 mm in any of the *x*, *y* or *z* directions, or rotations greater than 3 degrees, were excluded from further analysis. For each participant, probabilistic masks (ranging from 0 to 1) representing GM, WM and CSF were generated by segmenting the T_1_ structural images. Additionally, the structural images and tissue maps were normalized to the standard Montreal Neurological Institute space using the Diffeomorphic Anatomical Registration Through Exponentiated Lie Algebra (DARTEL) tool.^41^ This normalization was performed with a voxel size of 1.5 × 1.5 × 1.5 mm^3^. Nuisance signals, such as the Friston 24 head motion parameters obtained from realignment^42^ and the mean CSF time series extracted from a CSF brain mask generated through segmentation, were regressed out from the time course of each voxel. Finally, the voxel-wise time courses extracted from the normalized fMRI images were corrected for signal drifts and temporally filtered with a bandpass filter (0.01–0.1 Hz).

### White matter engagement analysis

The process of generating WM engagement maps follows the procedure described by Li *et al*.^35^ Firstly, we utilized a group-based averaged template created through the DARTEL tool after 18 iterative processes^41^ to create the group-based GM and WM masks. For the GM mask, a relatively lenient threshold (>0.6) was applied to the DARTEL-generated GM probability image, intended to ensure the comprehensive inclusion of GM voxels within the mask. Meanwhile, the WM mask was created using a stricter threshold (>0.95) on the WM probability image, ensuring that the areas labelled as WM are anatomically accurate and largely unaffected by partial volume effects from surrounding GM. Subsequently, we applied the thresholded WM and GM masks to the fMRI brain images of each participant.

The regions of interest are derived from the DMN, which has been found to be relevant to the *APOE4* allele and closely associated with memory functions.^24^ These regions were derived from the Human Brainnetome Atlas,^43^ which includes 246 regions corresponding to the 17 networks identified by Yeo *et al*.^44^ Specifically, we focused on three subsets of these networks, namely, A, B and C, of the DMN, which together encompass a total of 38 regions (Supplementary Fig. 1). Default-A contains the classically labelled DMN regions such as the prefrontal cortex, posterior cingulate cortex and angular gyri.^45^ Default-B and Default-C, while closely related to Default-A, exhibit unique connectivity patterns both within the DMN and with external networks. Default-B comprises the dorsomedial prefrontal cortex, the anterior inferior parietal lobule/temporoparietal junction and the lateral temporal cortex. Default-C, on the other hand, includes the cingulate gyrus, parahippocampal gyrus and hippocampus.^44^

For each individual, the mean time series was computed by averaging the fMRI signals across all voxels within each of the 38 DMN regions. Next, pairwise Pearson's correlation coefficients were computed between the mean time series of all region pairs. This produced a symmetric 38 × 38 full functional connectivity matrix M for each individual, representing the temporal coupling strength between DMN regions. What's more, to assess connectivity independent of local WM signals, we generated partial correlation matrices (*M*′*ₓ*) using each WM voxel (*x*)'s time series as a control. Each voxel's signal was averaged over its 5 × 5 × 5 WM neighbourhood (WM mask verified) to boost SNR prior to analysis.

**Figure 1:**
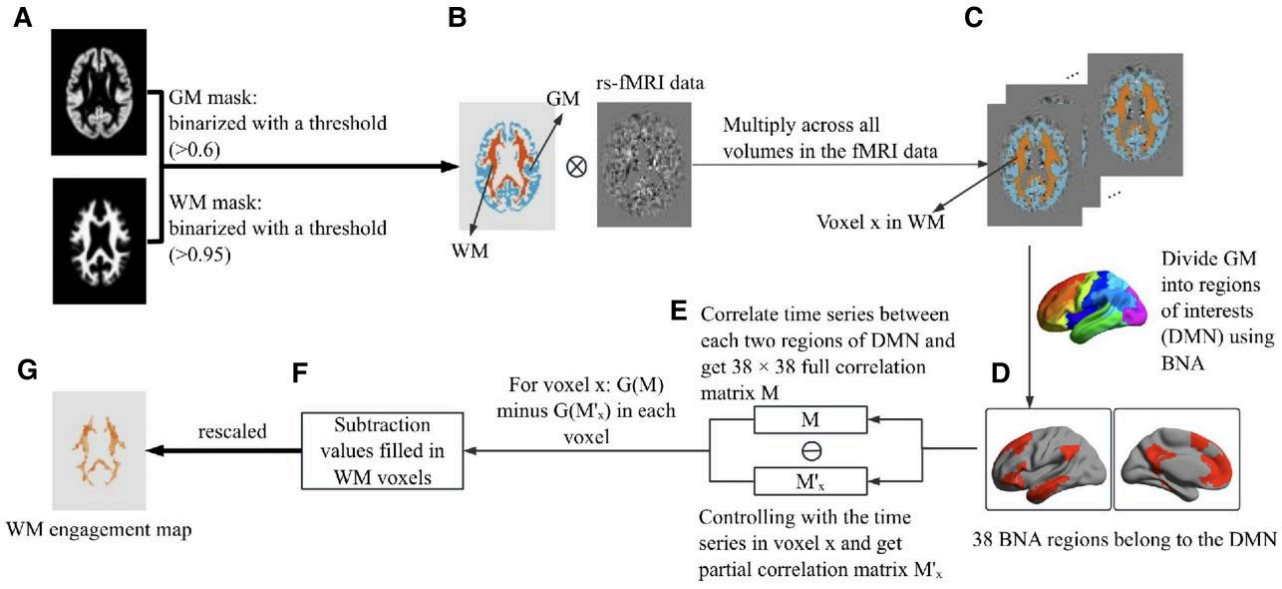
**Schematic representation of the overall pipeline of the algorithm of WM engagement maps.** (**A**) For each participant, group-based GM and WM masks were created using a template generated by the DARTEL tool. We applied a lenient threshold (>0.6) to the GM probability image to inclusively capture GM voxels, while a stricter threshold (>0.95) was used for the WM mask to ensure anatomical accuracy. (**B** and **C**) These thresholded WM and GM masks were then applied to the fMRI brain images. (**D**) The mean time series was computed by averaging the fMRI signals across all voxels within each of the 38 DMN regions. (**E**) Pairwise Pearson's correlation coefficients were calculated between the mean time series of all region pairs. In addition, to assess connectivity independent of local WM signals, we generated partial correlation matrices (*M'ₓ*) using the time series of each WM voxel (*x*) as a control. (**F**) Global connectivity metrics were computed by averaging the values from both the full correlation matrix (G(M)) and each partial correlation matrix (*G*(*M'ₓ*)). The difference between these metrics (Δ*Gₓ* = *G*(*M*) − *G*(*M'ₓ*)) was mapped across all WM voxels to create preliminary engagement maps. (**G**) The values on the WM engagement map were standardized using *Z*-scoring to normalize the data distribution for subsequent group-level analyses. GM, grey matter; WM, white matter; rs-fMRI, resting-state functional magnetic resonance imaging; DMN, default mode network; BNA, Brainnetome Atlas; M, 38 × 38 full functional connectivity matrix; *M'ₓ*, partial correlation matrices using each WM voxel (*x*)'s time series as a regressor; *G*(*M*), mean values of the full correlation matrix *M*; *G*(*M'ₓ*), mean value of the partial correlation matrix.

To assess WM engagement, we first computed global connectivity metrics by taking the mean values of both the full correlation matrix (*G*(*M*)) and each partial correlation matrix (*G*(*M*′*ₓ*)), where *M* controlled for the signal from WM voxel *x*. The difference between these metrics (Δ*Gₓ* = *G*(*M*) − *G*(*M*′*ₓ*)) was mapped across all WM voxels to generate preliminary engagement maps, reflecting each voxel's global network influence. Finally, we standardized the WM engagement map values using *Z*-scoring (subtracting the global mean and dividing by the standard deviation) to normalize the data distribution for subsequent group-level analyses. The Johns Hopkins University White Matter Tractography Atlas^46^ was applied to identify the regions of WM engagement changes. This entire process is illustrated in Fig. 1.

### Statistical analysis

Statistical analysis was performed using SPSS version 29.0.2.0 (SPSS, Inc., Chicago, IL, USA). A significance level was set at *P* < 0.05 for all analyses. *χ*^2^ test was employed to assess sex and group differences in study participant characteristics. Descriptive variables were reported as mean ± standard deviation and evaluated using a two independent samples *t*-test. Changes in participants' behavioural performance were calculated as Visit 2–Visit 1. ANCOVA was utilized to detect differences between low and high-risk groups in the calculated changes in memory score, adjusted for the baseline scores (Visit 1) of the primary memory measures mentioned in Table 1. The subgroup analysis was performed to examine potential sex differences in behavioural performance and WM engagement.

For the WM engagement maps derived from fMRI data, *within-group* comparisons involved separately analysing the changes (i.e. Visit 2–Visit 1) for both the low-risk and high-risk groups. A paired *t*-test was used to assess changes over time. *Between-group* comparisons were conducted by calculating changes in WM engagement across all voxels in the brain by subtracting WM engagement maps at Visit 1 from WM engagement maps at Visit 2.

Participants were stratified by *APOE* genotype and biological sex into four groups: high-risk females, low-risk females, high-risk males and low-risk males. Comparisons were made between

1. Females in the low-risk group versus females in the high-risk group
2. Males in the low-risk group versus males in the high-risk group
3. Low-risk females versus low-risk males
4. High-risk females versus high-risk males

Differences in changes in the WM engagement maps between these groups were analysed using a two-sample *t*-test. Results were corrected for multiple comparisons using GRF methods, with a cluster-level *P* < 0.05, a voxel-level *P* < 0.01 and a minimum cluster size of more than 10 voxels.

To investigate the relationship between behavioural and neuroimaging findings, partial Pearson’s correlation analysis was employed. The data were segmented into high- and low-risk groups for *within-group* comparisons. This analysis examined the correlation between changes in WM engagement maps (Visit 2–Visit 1) and behavioural measures, adjusting for sex and years of education. For *between-group* comparisons, the data were divided into female and male groups. Partial correlation between differences in WM engagement between low-risk and high-risk groups and behavioural measures was performed. Age and years of education were used as covariates.

Both age and sex significantly impact memory, a well-documented factor in cognitive research.^3,4^ Years of education is a proxy of cognitive reserve.^47^ In our previous study,^32^ we observed notable interaction effects between years of education and WM hyperintensities, as well as cerebral microbleeds, which affect inter-network structural and functional coupling. Therefore, we included these variables as covariates.

## Results

### Demographical and neuropsychological results

A total of 173 participants with a complete set of both visits, genotyping and fMRI data from the HABS were included in this analysis. Table 1 displays the demographic information and participant performance on the neuropsychological assessments. Briefly, the average age of the 173 participants was 73.15 years (SD: 6.2), with 121 participants in the high-risk group averaging 73.26 years (6.45) and 52 in the low-risk group averaging 72.91 years (5.62). No significant differences in age, sex or educational years were found between groups, nor were there significant changes in the listed measures at Visit 2.

For the non-primary neuropsychological features at Visit 1, a significant group difference in performance was observed on the Structured Telephone Interview of Dementia Assessment. Compared with the low-risk group, the high-risk group performed better (1.01 ± 1.11 and 1.5 ± 1.37 for low- and high-risk groups, respectively; *t* = −2.48, *P* = 0.014).

Regarding our primary outcome measures of interest, significant baseline differences (at Visit 1) were observed in the Selective Reminding Test—short-term retrieval (SRT_str). Notably, compared with the low-risk group, those in the high-risk group performed significantly worse (14.01 ± 5.63 and 16.63 ± 5.91 for low- and high-risk groups, respectively; *t* = −2.77, *P* = 0.006; shown in Table 3). The results indicated a significant decrease in SRT_str scores in the high-risk group compared to the low-risk group, with changes of −0.82 ± 5.596 and −4.15 ± 7.09 for the low- and high-risk groups, respectively (Table 4; *t* = 5.13, *P* = 0.025).

**Table 3:** Comparison of primary cognitive outcomes between high-risk and low-risk groups at baseline and follow-up

Variables	Visit 1				Visit 2			
	Non-APOE4 (*N* = 121)	APOE4 (*N* = 52)	*t*	*P*	Non-APOE4 (*N* = 121)	APOE4 (*N* = 52)	*t*	*P*
BNT	27.81 ± 2.64	28.12 ± 1.89	−0.76	0.451^a^	27.68 ± 2.92	28.1 ± 2.29	−0.91	0.366^a^
Digits_F_B (*n*)	1.83 ± 1.14	2.02 ± 1.13	−0.98	0.330^a^	1.88 ± 1.14	1.73 ± 1.19	0.76	0.448^a^
FCsrt_FNC (*n*)	47.68 ± 0.73	47.68 ± 1.18	0.03	0.980^a^	47.6 ± 0.82	47.46 ± 1.13	0.87	0.385^a^
FCsrt_Free (*n*)	33.71 ± 5.37	33.72 ± 5.92	−0.02	0.985^a^	33.71 ± 6.43	32.15 ± 7.41	1.39	0.165^a^
LetterNum_Total (score)	9.91 ± 2.35	9.49 ± 2.56	1.05	0.296^a^	10.01 ± 2.69	10.06 ± 2.6	−0.11	0.911^a^
LogisticMem_IL (score)	15.25 ± 3.18	14.96 ± 2.93	0.56	0.579^a^	17.21 ± 3.26	17.21 ± 3.51	−0.01	0.993^a^
LogisticMem_DR (score)	14 ± 3.15	13.88 ± 2.77	0.23	0.819^a^	16.12 ± 3.47	15.92 ± 3.94	0.34	0.738^a^
SRT_dr (*n*)	5.57 ± 2.93	5.02 ± 3.01	1.13	0.262^a^	6.61 ± 3.05	6.04 ± 3.62	1.07	0.286^a^
SRT_cltr (*n*)	19.02 ± 13.26	18.04 ± 13.43	0.44	0.658^a^	23.39 ± 15.33	24 ± 15.89	−0.24	0.812^a^
SRT_cr (*n*)	20.26 ± 10.95	18.42 ± 10.66	1.02	0.308^a^	23.87 ± 12.09	24.46 ± 13.05	−0.29	0.773^a^
SRT_ltr (*n*)	29.64 ± 13.39	26.83 ± 13.25	1.27	0.206^a^	33.4 ± 14.54	34.4 ± 16.34	−0.4	0.690^a^
SRT_lts (*n*)	32.85 ± 13.8	29.29 ± 13.64	1.56	0.120^a^	36.36 ± 14.54	37.6 ± 16.7	−0.47	0.643^a^
SRT_mc (*n*)	11.35 ± 1.02	11.15 ± 1.41	1.01	0.312^a^	11.62 ± 0.69	11.5 ± 0.98	0.92	0.359^a^
SRT_str (*n*)	14.01 ± 5.63	16.63 ± 5.91	−2.77	0.006^a^,**	13.19 ± 5.96	12.48 ± 6.83	0.69	0.493^a^
SRT_tr (*n*)	43.63 ± 9.07	43.46 ± 8.38	0.11	0.910^a^	46.6 ± 9.7	46.88 ± 10.43	−0.18	0.861^a^

BNT, Boston Naming Test; Digits_F_B, Digit Span Test (forward minus ackward); FCsrt_FNC, Free and Cued Selective Reminding Test—total of free and cued recall; FCsrt_Free, Free and Cued Selective Reminding Test—total of free recall; LetterNum_Total, Letter-Number Sequencing Test; LogisticMem_IL, Logical Memory—measure of immediate learning; LogicMem_DR, Logical Memory—measure of delayed recall; SRT_dr, Selective Reminding Test—delayed recall; SRT_cltr, Selective Reminding Test—continuous long-term retrieval; SRT_cr, Selective Reminding Test—continuous retrieval; SRT_ltr, Selective Reminding Test—long-term retrieval; SRT_lts, Selective Reminding Test—long-term storage; SRT_mc, Selective Reminding Test—recognition recall; SRT_str, Selective Reminding Test—short-term retrieval; SRT_tr, Selective Reminding Test—total recall.

^a^Two independent samples *t*-test.

**P < 0.01.

**Table 4:** Primary cognitive outcomes, changes in assessment scores between visits (second–first) for low- and high-risk groups (controlling for baseline scores)

Variables	Non-*APOE4* Second–first (*N* = 121)	*APOE4* Second–first (*N* = 52)	Between low- and high-risk groups	
			*F* value	*P* value
BNT	−0.12 ± 1.69	−0.02 ± 1.71	0.25	0.617^a^
Digits_F_B	0.04 ± 1.41	−0.29 ± 1.45	1.03	0.313^a^
FCsrt_FNC	−0.05 ± 0.78	−0.17 ± 1.39	0.85	0.359^a^
FCsrt_Free	0.46 ± 5.36	−1.26 ± 6.19	3.28	0.072^a^
LetterNum_Total	0.12 ± 2.28	0.53 ± 2.13	0.59	0.442^a^
LogisticMem_IL	1.96 ± 2.85	2.25 ± 3.12	0.17	0.680^a^
LogisticMem_DR	2.12 ± 3.06	2.04 ± 4.1	0.07	0.799^a^
SRT_dr	1.04 ± 2.66	1.02 ± 2.84	0.23	0.635^a^
SRT_cltr	4.37 ± 12.85	5.96 ± 12.23	0.43	0.514^a^
SRT_cr	3.6 ± 9.39	6.04 ± 9.85	1.71	0.193^a^
SRT_ltr	3.77 ± 11.12	7.58 ± 12.21	2.95	0.088^a^
SRT_lts	3.5 ± 11.63	8.31 ± 13.32	3.89	0.050^a^
SRT_mc	0.27 ± 1.08	0.35 ± 1.47	0.45	0.503^a^
SRT_str	−0.82 ± 5.6	−4.15 ± 7.09	5.13	0.025^a,^*
SRT_tr	2.97 ± 7.45	3.42 ± 6.9	0.13	0.720^a^

BNT, Boston Naming Test; Digits_F_B, Digit Span Test (forward minus backward); FCsrt_FNC, Free and Cued Selective Reminding Test—total of free and cued recall; FCsrt_Free, Free and Cued Selective Reminding Test—total of free recall; LetterNum_Total, Letter-Number Sequencing Test; LogisticMem_IL, Logical Memory—measure of immediate learning; LogicMem_DR, Logical Memory—measure of delayed recall; SRT_dr, Selective Reminding Test—delayed recall; SRT_cltr, Selective Reminding Test—continuous long-term retrieval; SRT_cr, Selective Reminding Test—continuous retrieval; SRT_ltr, Selective Reminding Test—long-term retrieval; SRT_lts, Selective Reminding Test—long-term storage; SRT_mc, Selective Reminding Test—recognition recall; SRT_str, Selective Reminding Test—short-term retrieval; SRT_tr, Selective Reminding Test—total recall.

^a^Analysis of covariance (ANCOVA).

^*^ *P* < 0.05.

When further stratified by biological sex and by *APOE4* (Table 5):

1. Females in low-risk versus high-risk: the Free and Cued Selective Reminding Test—total of free recall (FCsrt_Free) performance was significantly poorer in the high-risk group compared with the low-risk group (0.07 ± 5.68 and −3 ± 6.58 for low- and high-risk group, respectively; *t* = 5.69, *P* = 0.019).
2. Males in low-risk versus high-risk: the BNT showed a significant increase in scores between the two visits for the high-risk group compared to the low-risk group (−0.37 ± 1.5 and 0.48 ± 1.33 for low- and high-risk group, respectively; *t* = 4.52, *P* = 0.038). Additionally, in the high-risk male group, there were significant increases of the difference scores in three metrics of the SRT: continuous retrieval (low-risk: 1.28 ± 8.05, high-risk: 6.81 ± 10.05, *t* = 5.74, *P* = 0.02), long-term retrieval (low-risk: 1.16 ± 10.47, high-risk: 8.81 ± 13.07, *t* = 6.16, *P* = 0.016) and long-term storage (low-risk: 1.09 ± 11.53, high-risk: 9.48 ± 14.3, *t* = 5.97, *P* = 0.018). Conversely, a significant decrease of difference scores was observed in the short-term retrieval of SRT for the high-risk group compared to the low-risk group (0.3 ± 5.54 and −4.86 ± 7.29 for low- and high-risk group, respectively; *t* = 7.58 and *P* = 0.008).
3. Low-risk females versus males: in the low-risk male group, there was a notable decrease in difference scores compared to females across the immediate learning of Logical Memory Test (female: 2.23 ± 2.6, male: 1.47 ± 3.23, *t* = 4.17, *P* = 0.043) and six metrics of SRT: delayed recall (female: 1.33 ± 2.62, male: 0.51 ± 2.68, *t* = 8.55, *P* = 0.004), continuous long-term retrieval (female: 6.04 ± 13.48, male: 1.35 ± 11.14, *t* = 8.18, *P* = 0.005), continuous retrieval (female: 4.88 ± 9.87, male: 1.28 ± 8.05, *t* = 9.27, *P* = 0.003), long-term retrieval (female: 5.21 ± 11.27, male: 1.16 ± 10.47, *t* = 8.62, *P* = 0.004), long-term storage (female: 4.83 ± 11.55, male: 1.09 ± 11.53, *t* = 7.89, *P* = 0.006) and total recall (female: 3.79 ± 7.33, male: 1.47 ± 7.51, *t* = 7.09, *P* = 0.009). Conversely, a significant increase of difference scores in the short-term retrieval of the SRT was observed in the male group compared to the female group (−1.44 ± 5.57 and 0.3 ± 5.54 for female and male group, respectively; *t* = 8.06 and *P* = 0.005).
4. High-risk females versus males: only the FCsrt_Free presented a significant increase in the scores between the two visits for the male group compared to the female group (−3 ± 6.58 and 1.32 ± 4.62 for female and male group, respectively; *t* = 5.15, *P* = 0.028).

**Table 5:** Primary cognitive outcomes, score changes between visits (second–first) for sex and risk groups (female and male; low-risk and high-risk), controlling for baseline scores

Variables	Female Non-*APOE4* Second–first (*N* = 78)	Female *APOE4* Second–first (*N* = 31)	Male Non-*APOE4* Second–first (*N* = 43)	Male *APOE4* Second–first (*N* = 21)	Females Between low- and high-risk groups		Males Between low- and high-risk groups		Non-*APOE4* Between female and male groups		*APOE4* Between female and male groups	
					*F* value	*P* value	*F* value	*P* value	*F* value	*P* value	*F* value	*P* value
BNT	0.01 ± 1.78	−0.35 ± 1.87	−0.37 ± 1.5	0.48 ± 1.33	0.69	0.409^a^	4.52	0.038^a^,*	0.69	0.408	3.73	0.059
Digits_F_B	0.08 ± 1.42	−0.35 ± 1.28	−0.02 ± 1.39	−0.19 ± 1.69	1.53	0.218^a^	0.02	0.882^a^	0.04	0.85	0.62	0.434
FCsrt_FNC	−0.08 ± 0.8	−0.18 ± 1.74	0.03 ± 0.75	−0.16 ± 0.6	0.64	0.425^a^	0.73	0.396^a^	0.42	0.52	0.08	0.777
FCsrt_Free	0.07 ± 5.68	−3 ± 6.58	1.18 ± 4.68	1.32 ± 4.62	5.69	0.019^a,^*	0.02	0.886^a^	0.18	0.672	5.15	0.028*
LetterNum_Total	0.24 ± 2.29	0.67 ± 2.26	−0.09 ± 2.29	0.33 ± 1.96	0.3	0.586^a^	0.88	0.353^a^	0.67	0.415	0.02	0.89
LogisticMem_IL	2.23 ± 2.6	2.23 ± 3.1	1.47 ± 3.23	2.29 ± 3.24	0.02	0.894^a^	0.25	0.620^a^	4.17	0.043*	0.49	0.487
LogisticMem_DR	2.4 ± 3.05	2.1 ± 4.21	1.63 ± 3.05	1.95 ± 4.02	0.12	0.735^a^	0.02	0.880^a^	3.78	0.054	0.76	0.386
SRT_dr	1.33 ± 2.62	1.39 ± 3.01	0.51 ± 2.68	0.48 ± 2.54	0.35	0.557^a^	0.001	0.979^a^	8.55	0.004**	1.43	0.238
SRT_cltr	6.04 ± 13.48	5.13 ± 11.57	1.35 ± 11.14	7.19 ± 13.33	0.22	0.640^a^	3.63	0.061^a^	8.18	0.005**	0.10	0.752
SRT_cr	4.88 ± 9.87	5.52 ± 9.84	1.28 ± 8.05	6.81 ± 10.05	<0.001	0.998^a^	5.74	0.020^a,^*	9.27	0.003**	0.07	0.796
SRT_ltr	5.21 ± 11.27	6.74 ± 11.75	1.16 ± 10.47	8.81 ± 13.07	0.11	0.742^a^	6.16	0.016^a,^*	8.62	0.004**	0.16	0.693
SRT_lts	4.83 ± 11.55	7.52 ± 12.79	1.09 ± 11.53	9.48 ± 14.3	0.43	0.515^a^	5.97	0.018^a,^*	7.89	0.006**	0.08	0.773
SRT_mc	0.27 ± 0.98	0.45 ± 1.55	0.28 ± 1.26	0.19 ± 1.37	0.01	0.920^a^	0.49	0.486^a^	1.79	0.183	1.63	0.208
SRT_str	−1.44 ± 5.57	−3.68 ± 7.03	0.3 ± 5.54	−4.86 ± 7.29	0.54	0.465^a^	7.58	0.008^a,^**	8.06	0.005**	0.15	0.696
SRT_tr	3.79 ± 7.33	3.06 ± 6.19	1.47 ± 7.51	3.95 ± 7.97	0.29	0.593^a^	1.78	0.188^a^	7.09	0.009**	0.12	0.734

BNT, Boston Naming Test; Digits_F_B, Digit Span Test (forward minus backward); FCsrt_FNC, Free and Cued Selective Reminding Test—total of free and cued recall; FCsrt_Free, Free and Cued Selective Reminding Test—total of free recall; LetterNum_Total, Letter-Number Sequencing Test; LogisticMem_IL, Logical Memory—measure of immediate learning; LogicMem_DR, Logical Memory—measure of delayed recall; SRT_dr, Selective Reminding Test—delayed recall; SRT_cltr, Selective Reminding Test—continuous long-term retrieval; SRT_cr, Selective Reminding Test—continuous retrieval; SRT_ltr, Selective Reminding Test—long-term retrieval; SRT_lts, Selective Reminding Test—long-term storage; SRT_mc, Selective Reminding Test—recognition recall; SRT_str, Selective Reminding Test—short-term retrieval; SRT_tr, Selective Reminding Test—total recall.

^a^Analysis of covariance (ANCOVA).

^*^ *P* < 0.05, **P < 0.01.

### White matter engagement with the DMN

#### Within-group

**Figure 2:**
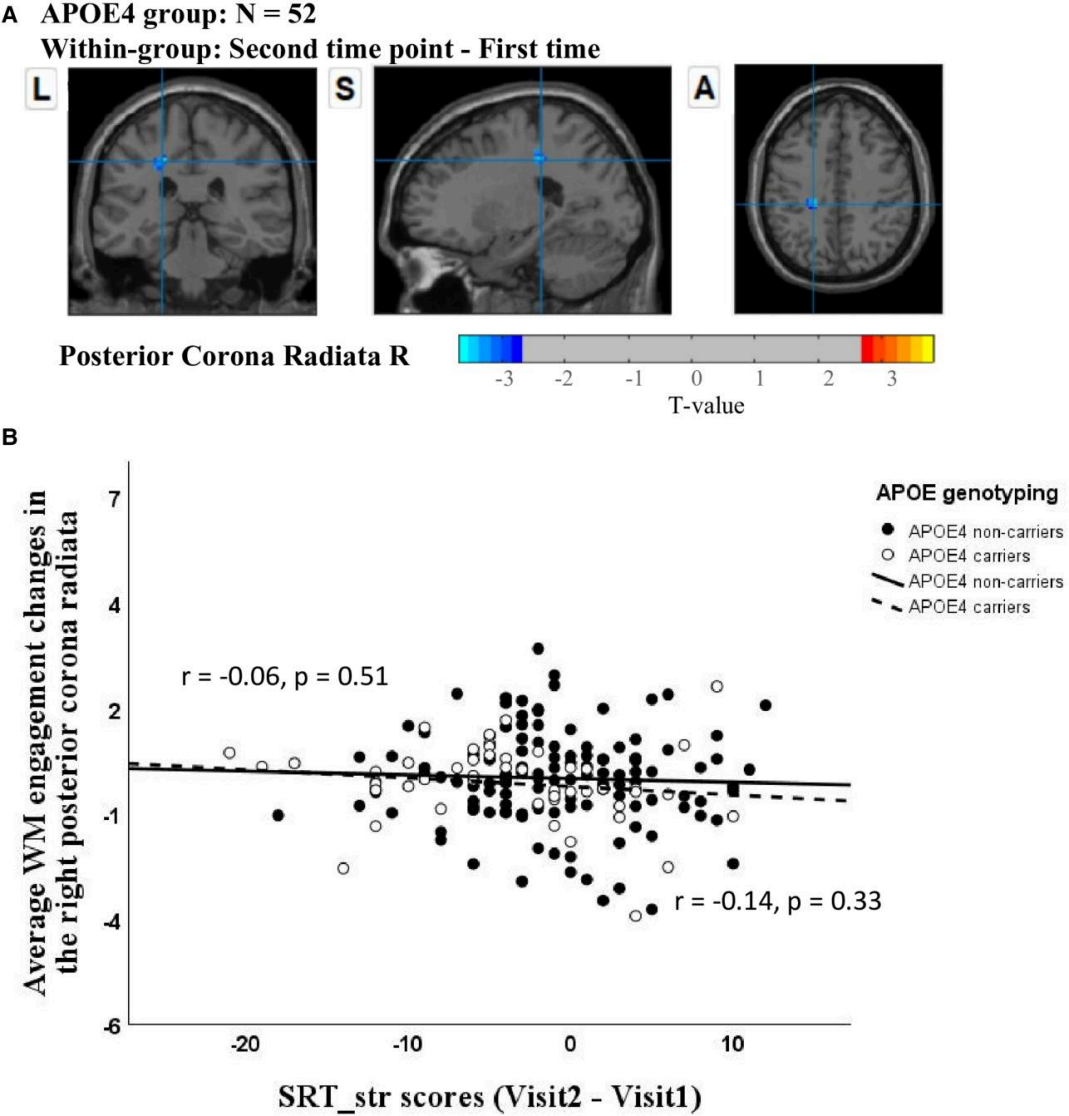
**Within-group comparison of WM engagement with the DMN across two time visits.** (**A**) Neuroimaging results from the study showing a comparison of WM engagement with the DMN between two visits (Visit 2–Visit 1) in the high-risk group, analysed using a paired *t*-test (*t* > 2.7, significance confirmed via GRF correction with cluster-level *P* < 0.05, voxel-level *P* < 0.01 and minimum cluster sizes exceeding 10 voxels). (**B**) Scatter plots depicting partial correlations between the average changes in WM engagement (Visit 2–Visit 1) in the right posterior corona radiata and the differences in SRT_str scores (Visit 2–Visit 1). The correlations are adjusted for sex and years of education and are presented for both high-risk and low-risk groups. WM, white matter; DMN, default mode network; SRT_str, Selective Reminding Test—short-term retrieval.

We observed a significant reduction in WM engagement of the DMN within the right posterior corona radiata across two visits (i.e. Visit 2–Visit 1) in the high-risk group (Fig. 2A). No significant changes were detected in the low-risk group over the two visits.

#### Between-group

**Figure 3:**
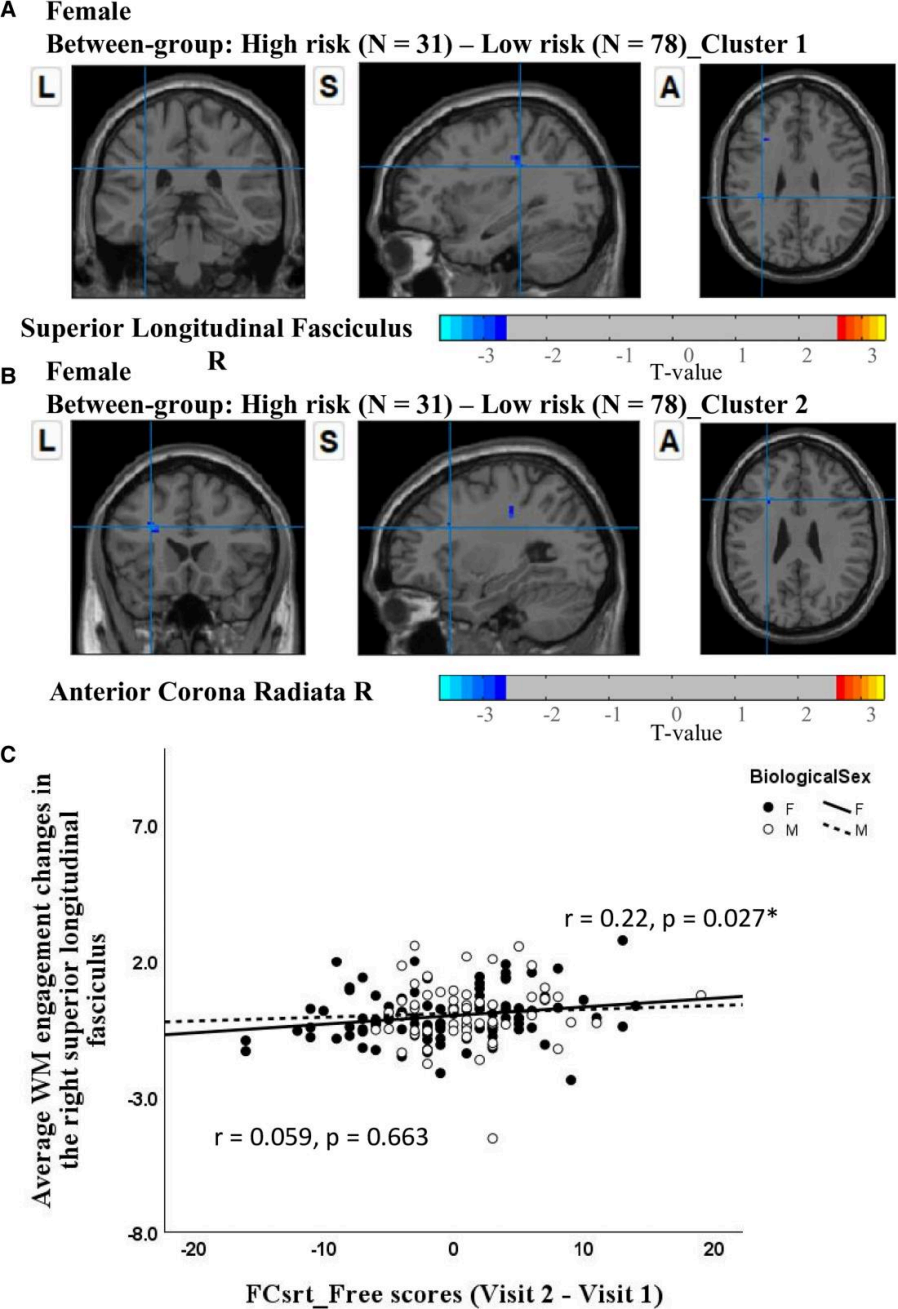
**Between-group differences in WM engagement with the DMN by genetic risk level in females.** Neuroimaging results from the study reveal two significant clusters identified through between-group comparisons (high-risk minus low-risk) in females using a two-sample *t*-test (*t* > 2.7, significance confirmed via GRF correction with cluster-level *P* < 0.05, voxel-level *P* < 0.01 and minimum cluster sizes exceeding 10 voxels): (**A**) Cluster 1 located in the right SLF and (**B**) in the right anterior corona radiata. (**C**) Scatter plots depicting partial correlations between the average WM engagement changes in the right SLF and FCsrt_Free scores (Visit 2–Visit 1), controlling for age and years of education, across female and male groups. WM, white matter; DMN, default mode network; FCsrt_Free, Free and Cued Selective Reminding Test—total of free recall.

**Figure 4:**
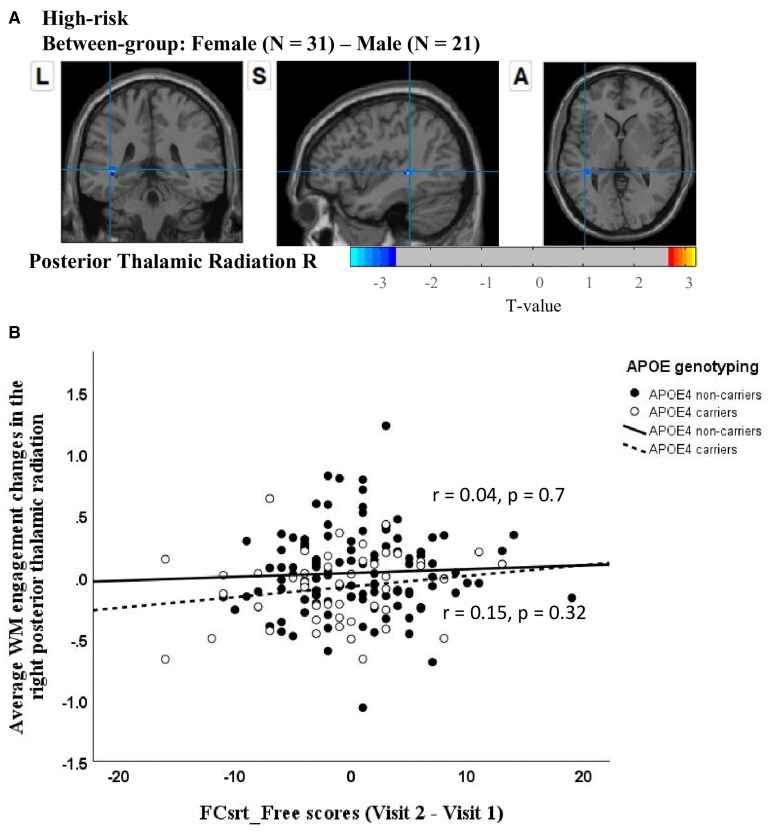
**Between-group differences in WM engagement with the DMN by sex in high-risk group.** (**A**) Neuroimaging results from the study reveal one significant cluster identified through between-group comparisons (female minus male) in high-risk group using a two-sample *t*-test (*t* > 2.7, significance confirmed via GRF correction with cluster-level *P* < 0.05, voxel-level *P* < 0.01 and minimum cluster sizes exceeding 10 voxels): located in the right posterior thalamic radiation. (**B**) Scatter plots depicting partial correlations between the average WM engagement changes in the right posterior thalamic radiation and FCsrt_Free scores (Visit 2–Visit 1), controlling for age and years of education, across high-risk and low-risk groups. WM, white matter; DMN, default mode network; FCsrt_Free, Free and Cued Selective Reminding Test—total of free recall.

Taking biological sex and *APOE* genotype into consideration:

1. Females in low-risk versus high-risk: our analysis comparing changes in WM engagement (Visit 2–Visit 1) revealed that, relative to females in the low-risk group, females in the high-risk group showed a significant reduction in WM engagement of the DMN within the right SLF and right anterior corona radiata (Fig. 3B and C).
2. Males in low-risk versus high-risk: no statistical differences were observed between the risk groups in males.
3. Low-risk females versus males: no statistical differences in WM engagement were observed between the females and males in the low-risk group.
4. High-risk females versus males: our analysis of changes in WM engagement (Visit 2–Visit 1) revealed that, compared to males in the high-risk group, females in the high-risk group exhibited a significant reduction in WM engagement within the DMN, specifically in the right posterior thalamic radiation (as shown in Fig. 4A).

### Relationship between cognitive outcomes and neuroimaging findings

#### Within-group

In the high-risk group, there was a negative but non-significant correlation (*r* = −0.14, *P* = 0.33) between changes in WM engagement with the DMN in the right posterior corona radiata and performance difference on the SRT_str across visits (Visit 2–Visit 1), after controlling for sex and years of education. This suggests a potential link where reduced WM engagement of the DMN in the right posterior corona radiata (i.e. Visit 2–Visit 1) could be associated with increased reliance on short-term retrieval on the SRT (indicating poorer performance) at the follow-up assessment. Similarly, in the low-risk group, no significant correlations were found between changes in WM engagement of the DMN and short-term retrieval performance in the low-risk group (*r* = −0.06, *P* = 0.51) (Fig. 2B).

#### Between-group differences

1. Females in low-risk versus high-risk: the change (Visit 2–Visit 1) of WM engagement map of the DMN in the right SLF among females was positively correlated with the change in performance (i.e. Visit 2–Visit 1) on the FCsrt_Free (*r* = 0.22, *P* = 0.027), after controlling for age and years of education (Fig. 3C). In other words, relative to females in the low-risk group, reduced WM engagement of the DMN in the right SLF was associated with diminished free recall performance (female low-risk: 0.07 ± 5.68, female high-risk: −3 ± 6.58, *t* = 5.69, *P* = 0.019; Table 5) on the FCsrt among females in the high-risk group.
2. Males in low-risk versus high-risk: no significant correlations were observed in males (*r* = 0.06, *P* = 0.663) (Fig. 3B).
3. Low-risk females versus males: no significant correlations were observed in the low-risk group.
4. High-risk females versus males: no significant correlation was found with the change in performance (i.e. Visit 2–Visit 1) on the FCsrt_Free, after controlling for age and years of education (Fig. 4B).

## Discussion

This study evaluated longitudinal behavioural and neuroimaging changes, comparing them across sex and risk groups. We observed a significant decline in SRT_str and reduced WM engagement in the DMN within the right posterior corona radiata, exclusively in the high-risk group. This reduction was negatively correlated with the performance difference across visits. Additionally, while high-risk males showed improvements in various cognitive tests (BNT and measures of SRT), no differences in WM engagement of the DMN were noted compared to low-risk males. In contrast, high-risk females exhibited worse performance on the FCsrt_Free test and reduced WM engagement in the right SLF compared to low-risk females; this reduction positively correlated with changes in recall performance between visits. In comparisons within the low-risk group, males demonstrated poorer performance than females on several memory tests (LogisticMem_IL and measures of SRT), whereas high-risk males only showed significant improvements on the FCsrt_Free compared to high-risk females. No significant neuroimaging correlations with cognitive changes were found in high-risk groups across sexes.

Behaviourally, we focused on memory-related outcome variables. The BNT assesses semantic memory, while the Digit Span Test measures working memory capacity. The FCsrt_Free represents a test of verbal episodic memory, and the LetterNum is another measure of working memory. Additionally, the Logical Memory Test and the SRT both evaluate aspects of verbal memory. In the high-risk group at baseline, a higher score in short-term retrieval was observed, indicating potential difficulty in transferring items from short-term to long-term memory. A higher score in this metric suggests that while participants may effectively recall information immediately, they may struggle to encode this information into long-term storage.^48^ A previous large cohort study involving 3257 healthy men and women aged 18–94 years suggested that there exists a significant linear association between age and SRT performance, where poorer total recall score was correlated with advancing age.^49^ Findings from this study also demonstrated that men, relative to women, displayed greater dependence on STS/STR as they age. Similar results were reported in other normative data sets.^50,51^ Contrary to these results, within the HABS data set, we found that in both males and females, regardless of *APOE* status, there was an overall improvement in SRT performance across time in both high- and low-risk groups. This shift in memory storage and retrieval paradigm was particularly evident among high-risk males, who showed a statistically significant reduction in STR and improvements in CLTR, CR, LTS and LTR across time compared with low-risk males. This suggests that, within the context of this data set, the male *APOE4* carriers may be more resilient against the effects of this risk factor. However, future studies are necessary to confirm this proposition.

Additionally, over the 3-year span from Visit 1 to Visit 2, regardless of sex, none of the *APOE4* carriers progressed to develop worse cognitive function or memory compared with non-carriers in the majority of the tests examined in this study, aside from FCsrt free recall, in which female *APOE4* carriers declined significantly over time, while male *APOE4* carriers maintained their level of performance. The fact that females are more impacted by Alzheimer's disease pathology is widely supported by literature.^7,52^ This aligns with evidence that concluded there exist an sex-by-*APOE4* interaction where females with the *APOE4* allele have four times greater risk for subsequent cognitive decline and developing Alzheimer's disease than females without, or men with the *APOE4* allele.^53^ Greater decline in memory as well as executive functions in female carriers of *APOE4*, compared to male carriers, is even more evident when compounded with presence of other Alzheimer's disease pathology (e.g. tau, amyloid beta) and lower education attainment.^10,54^

There also appears to be cognitive benefits correlated with *APOE4* carriership. Past and recently studies have demonstrated that *APOE4* may enhance memory performance, particularly in verbal and working memory.^55,56^ Similarly, earlier study that showed younger *APOE4* carriers exhibited better cognitive control from more efficient neural resource allocation.^57^ These findings align with the apolipoprotein E compensatory mechanism recruitment hypothesis, which posited that the improved cognitive performance may be underpinned by greater levels of neural activation^58^ and recruitment of additional brain areas.^59^ This cognitive benefit and compensatory mechanism may be better retained in male than female *APOE4* carriers, suggesting male carriers may be more resilient to the negative impacts. The fact that our data showed female carriers declined faster than male carriers in cognitive performance may be indicative that the underlying compensatory mechanism was disrupted in the females—a concept that is illustrated and supported by our reported observation in sex-related differences in the WM engagement map.

For the neuroimaging results, when comparing data from the two time points (Visit 2–Visit 1), we observed a reduction in WM engagement with the DMN in the right posterior corona radiata in the high-risk group. This change in WM engagement was found to negatively correlate with the performance change on the SRT_str. The corona radiata, particularly its right posterior segment, is essential for transmitting motor and sensory information. In a study examining the impacts of ischaemic stroke in the corona radiata and pons, we observed increased degree centrality changes in the DMN, particularly involving the anterior cingulate cortex and posterior cingulate cortex. Additionally, the increased weighted importance of the DMN may suggest a compensatory mechanism aimed at maintaining coordination of information flow.^60^ Building on this finding, the DMN indeed has a close relationship with the corona radiata. Our study demonstrated that the right posterior corona radiata exhibited reduced engagement with the DMN over time in the high-risk group, and this alteration was associated with declines in verbal memory. However, in the Visit 2, the high-risk group presented improved short-term retrieval performance. To understand the contradiction, it is essential to analyse the differences between sexes in greater detail.

After stratifying the cohort into high-risk female, low-risk female, high-risk male and low-risk male groups, we observed a significant reduction in the engagement of the right posterior thalamic radiation with the DMN when comparing high-risk females to high-risk males. These findings highlight potential sex-specific neural mechanisms of risk in critical brain regions. Plenty of studies, as reported in references,^61,62^ have indicated that the thalamus may show signs of inflammation-related tissue swelling and/or myelin loss in individuals carrying the *APOE4* allele. An analysis of the effects of the *APOE4* allele on WM integrity, utilizing data from 28 494 British participants in the UK Biobank, revealed a significant impact on the sagittal stratum and posterior thalamic radiation in both men and women.^63^ The effects of the *APOE4* allele on the posterior thalamic radiation are related to the localized ventricular expansion, a hallmark feature of Alzheimer's disease pathology that correlates with cognitive decline.^64,65^ In particular, studies indicate that female *APOE4* carriers may experience greater hypometabolism and faster rates of atrophy in the thalamus, caudate nuclei, temporal lobes and occipital lobes compared to their male counterparts.^66,67^ These findings highlight a potential sex-dependent vulnerability of the cortico-thalamic feedback connections to *APOE4*-associated neurodegenerative processes. Expanding on previous structural or functional MRI research, our findings present that high-risk females are more vulnerable to cognitive decline and exhibit reduced engagement of posterior thalamic radiation, which involves functional brain activity between WM and the DMN in GM. It appears that high-risk males maintain intact connections in the posterior thalamic radiation with the DMN compared to high-risk females, and their cognitive performance is significantly better across time. However, the correlation is not significant, possibly due to an insufficient sample size, variability in the severity of risk factors or the presence of other confounding variables.

Also, we observed a noticeable reduction in engagement with the DMN in the right SLF and right anterior corona radiata in the high-risk female group compared to the low-risk female group. The *APOE4* allele has been shown to affect WM integrity in both young and elderly adults, with significant changes observed in widespread areas involving corona radiata and SLF compared to non-carriers.^68^ Operto *et al*.^22^ identified significant differences in SLF, which connects the frontal regions to the posterior brain regions (referred to DMN regions) between *APOE* carriers and non-carriers. Moreover, older participants (aged 65 and above) who were *APOE4* carriers exhibited higher mean diffusivity in the SLF and in the portion of the cingulum bundle adjacent to the cingulate cortex, compared to non-carriers.^69^ Notably, our study has directly linked reductions in SLF to engagement with the DMN, which correlates with a decline in episodic memory. These findings suggest that these changes could serve as potential biomarkers for memory function deterioration.

A recent study by Inglis^70^ highlighted that the *APOE4* allele significantly increases the risk of Alzheimer's disease, particularly among women. This increased risk is linked to impairments in microglial function, which are less effective at containing Alzheimer's disease pathology in *APOE4* carriers. Our study aligns with these findings, demonstrating that females in the low-risk group exhibited superior performance compared to those in the high-risk group. Additionally, we found that males in the low-risk group showed worse memory function than their high-risk counterparts. Interestingly, high-risk males performed better than high-risk females, although this pattern was not observed in the low-risk group. Similar to our findings, previous studies have also identified significant sex-related differences in *APOE4* carriers, with notable effects reported only in women.^71^ Lipid metabolism may underscore these sex differences in *APOE4* carriers. The impact of the *APOE4* allele on cholesterol and low-density lipoprotein metabolism is significantly altered in female Alzheimer's disease patients, but not in males.^72^ Modulations of endogenous sex hormones,^73^ particularly after menopause, may amplify the detrimental impact of *APOE4* in females due to the loss of oestrogen's protective effects.^74^ The absence of significant changes between WM and the DMN among males with the *APOE4* allele could further support the hypothesis of a relative resistance to the effects of the *APOE4* gene in males. Overall, our results, obtained using this novel technique, confirm the effectiveness and accuracy of the WM engagement maps.

There are several limitations to this study. First, the sample sizes for carriers and non-carriers, as well as females and males, are not equal. This discrepancy is challenging to address due to the varying prevalence of the *APOE* genotype and the differences in longevity between females and males. Second, fMRI does not provide a sufficiently high temporal resolution to accurately model the timing of neural events, and the scanning duration may be inadequate. Third, the thresholds we utilized for the probabilistic WM and GM masks might not be completely accurate. GM contamination can occur due to misclassification arising from image distortion.

## Conclusion

We explored the involvement of WM in the DMN and elucidated the relevance of this new metric to memory processes. The correlation between neuroimaging results and behavioural data further substantiates this approach. These findings enhance our understanding of the *APOE* genotype and emphasize the significance of sex-specific research in unravelling the neural mechanisms that underlie the risk and progression of Alzheimer's disease.

## Supplementary Material

fcaf278_Supplementary_Data

## Data Availability

The data that support the findings of this study are available from the corresponding author, upon reasonable request. Here is the link: https://polyuit-my.sharepoint.com/:f:/g/personal/huizhang_polyu_edu_hk/Enm0dWygEDdIiU0rtJMp2lUBi0Qvby3Y3UOIinG5S1nfvw?e=HVkgms.
