# Supplementary material for: Longitudinal effects of sex differences and apolipoprotein E genotype on white matter engagement among elderly
Source: Brain Commun. 2025 Jul 17;7(4):fcaf278. doi: 10.1093/braincomms/fcaf278 (PMC12308282; doi:10.1093/braincomms/fcaf278)
Supplement: fcaf278_Supplementary_Data [file fcaf278_supplementary_data.docx]

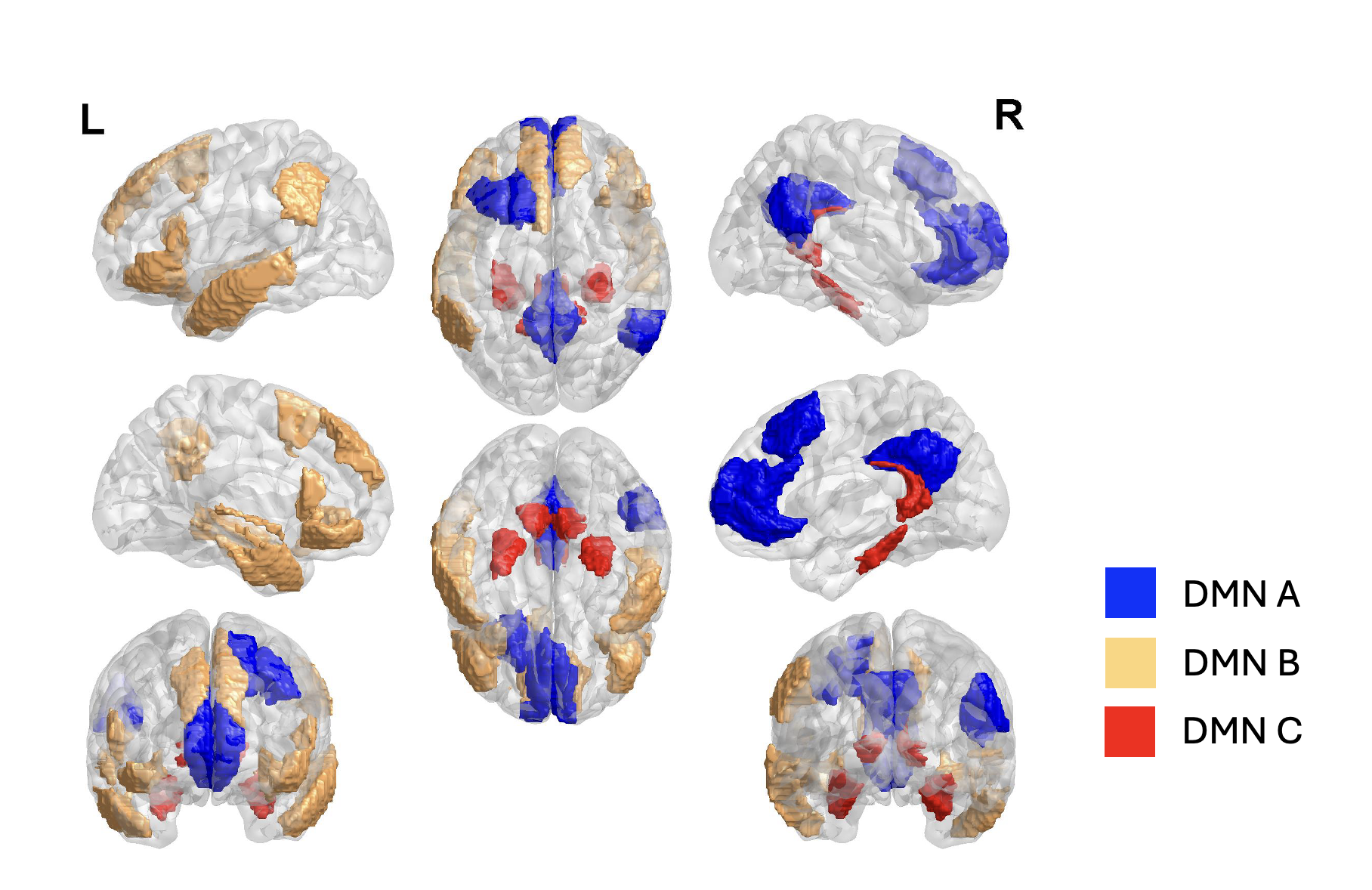


Supplementary Figure 1. This figure showcases the intricate subdivisions within the Default Mode Network (DMN). The figure was generated by our team using the BrainNet Viewer software (<https://www.nitrc.org/projects/bnv>) referring to the information from the Human Brainnetome Atlas (https://atlas.brainnetome.org/index.html), which encompasses 246 brain regions distributed across 17 distinct networks identified by Yeo et al^1^. Our analysis primarily centers on three critical subsets of these networks, designated as DMN A, DMN B, and DMN C, collectively comprising 38 unique regions.

Supplementary Reference:

1. Yeo BT, Krienen FM, Sepulcre J*, et al*. The organization of the human cerebral cortex estimated by intrinsic functional connectivity. *J Neurophysiol*. Sep 2011;106(3):1125-65. doi:10.1152/jn.00338.2011
